# Intrapleural targeted therapies (anti-VEGF and anti-EGFR) in the model of malignant pleural effusion

**DOI:** 10.18632/oncotarget.21362

**Published:** 2017-09-28

**Authors:** Milena Marques Pagliarelli Acencio, Juliana Puka, Vanessa Adélia Alvarenga, Vanessa Martins, Mariana Lombardi Peres de Carvalho, Evaldo Marchi, Vera Luiza Capelozzi, Lisete Ribeiro Teixeira

**Affiliations:** ^1^ Laboratorio de Pleura / LIM09 - Divisao de Pneumologia, Instituto do Coracao (InCor), Hospital das Clinicas HCFMUSP, Faculdade de Medicina, Universidade de Sao Paulo, Sao Paulo SP, Brazil; ^2^ Departmento de Patologia, Faculdade de Medicina, Universidade de Sao Paulo, Sao Paulo SP, Brazil; ^3^ Laboratorio de Genetica e Cardiologia Molecular, Instituto do Coracao (InCor), Hospital das Clinicas HCFMUSP, Faculdade de Medicina, Universidade de Sao Paulo, Sao Paulo SP, Brazil; ^4^ Faculdade de Medicina de Jundiai, Jundiai SP, Brazil

**Keywords:** targeted therapies, malignant pleural effusion, VEGF, EGFR, experimental model

## Abstract

**Rationale:**

Malignant pleural effusion has few options of treatment and drugs administrated by different routes can lead to a less permissive microenvironment for the development of malignant pleural disease.

**Objectives:**

To analyze therapies administered intrapleurally in malignant pleural disease and to study EGFR and KRAS mutations in adenocarcinoma.

**Methods:**

Mice received LLC cells and were treated intrapleurally with anti-VEGF, anti-EGFR, anti-VEGF+anti-EGFR or saline. Animal survival, weight and mobility, volume, biochemistry and immunology of fluid, gene expression, KRAS and EGFR mutation were evaluated.

**Results:**

All animals developed malignant effusion and presented progressive weight loss without difference between groups; however, groups treated with anti-EGFR were more active. No difference in mortality was observed. Temporal increase of volume and inflammatory markers was observed mainly in the untreated group. Gene expression in tumors was overexpressed in VEGF, EGFR and KRAS compared with normal tissue. Mutation in exon 2 of the KRAS gene was observed.

**Conclusions:**

Intrapleural Anti-VEGF and/or anti-EGFR reduced volume and inflammatory mediators in pleural fluid. Anti-EGFR and anti-VEGF+anti-EGFR decreased morbidity although without impact on survival. LLC tumors presented KRAS mutation, this could have influenced the action of these therapies.

## INTRODUCTION

Malignant pleural effusion mainly resulting from lung adenocarcinomas signals a disease of poor prognosis that is incurable by surgery, with impairment of quality of life and limited treatment that does not modify disease progression or benefit all patients [1, 2].

Studies have been developed to better the physiopathological understanding that allows for changes in the treatment of neoplastic pleural effusion, especially after the discovery of therapies directed at molecular targets [3, 4].

Better knowledge of tumor biology, especially of specific molecular alterations such as mutations of the epidermal growth factor receptor (EGFR) and the KRAS gene [3, 4] led to the discovery of the biomarkers used as target therapies. There are for example tyrosine kinase inhibitors and monoclonal antibodies against vascular endothelial growth factor (VEGF) and EGFR. These target therapies have discreetly improved overall survival as well as progression-free survival in patients with advanced disease. [3, 4]

VEGF is one of the main factors in the formation of malignant pleural effusion. It inhibits pleural defense mechanisms, allowing malignant cells to develop a vessel-rich environment for their nutrition thus facilitating tumor growth and the formation of pleural implants [5, 6].

EGFR is a tyrosine kinase receptor considered oncogenic and responsible for growth, survival, proliferation and differentiation of various cell types [3, 7]. It is altered in several types of tumors, especially in epithelial cells either by hyperexpression or mutations, inducing uncontrolled growth or malignant phenotype [8].

KRAS (Kirsten Rat Sarcoma) genes are key elements in the EGF-mediated signaling pathway, jointly regulating cell growth, differentiation and apoptosis through interaction with multiple effectors [9]. Their mutations are signs of poor prognosis and are related to a decreased response to EGFR inhibitor drugs, both in their extracellular portion (cetuximab) and in their tyrosine kinase domain (erlotinib and gefitinib) [10, 11].

The effects of chemotherapy on malignant pleural effusion are still uncertain but new drugs have gradually improved the prognosis for patients with lung cancer, with promising effects also in those with malignant effusion [12–14].

Experimental animal models mimic the human condition contributing to enhanced understanding of the mechanisms involved in malignant pleural fluid formation in humans and possible therapeutic strategies.

In 2006 Stathopoulos et al [15]. developed and described an experimental animal model for producing malignant pleural effusion. Lewis Lung Carcinoma (LLC) cells were injected directly into the pleural space of mice resulting in lung adenocarcinoma and malignant pleural effusion similar to that seen in humans.

Recent studies using this model have demonstrated the possibility of obtaining a less permissive microenvironment for the development of malignant pleural effusion by employing known drugs or a combination of chemotherapeutic agents administered intrapleurally or through other means at first detection of malignant pleural disease [7, 15–17].

Therefore, the objective of this study was to analyze the performance of targeted therapies in experimental models, administered alone or in combination via intrapleural route in different phases of malignant pleural disease. The study also evaluated the frequency of EGFR and KRAS mutations in the adenocarcinoma tumor cell line with LLC cells.

## RESULTS

**Figure 1:**
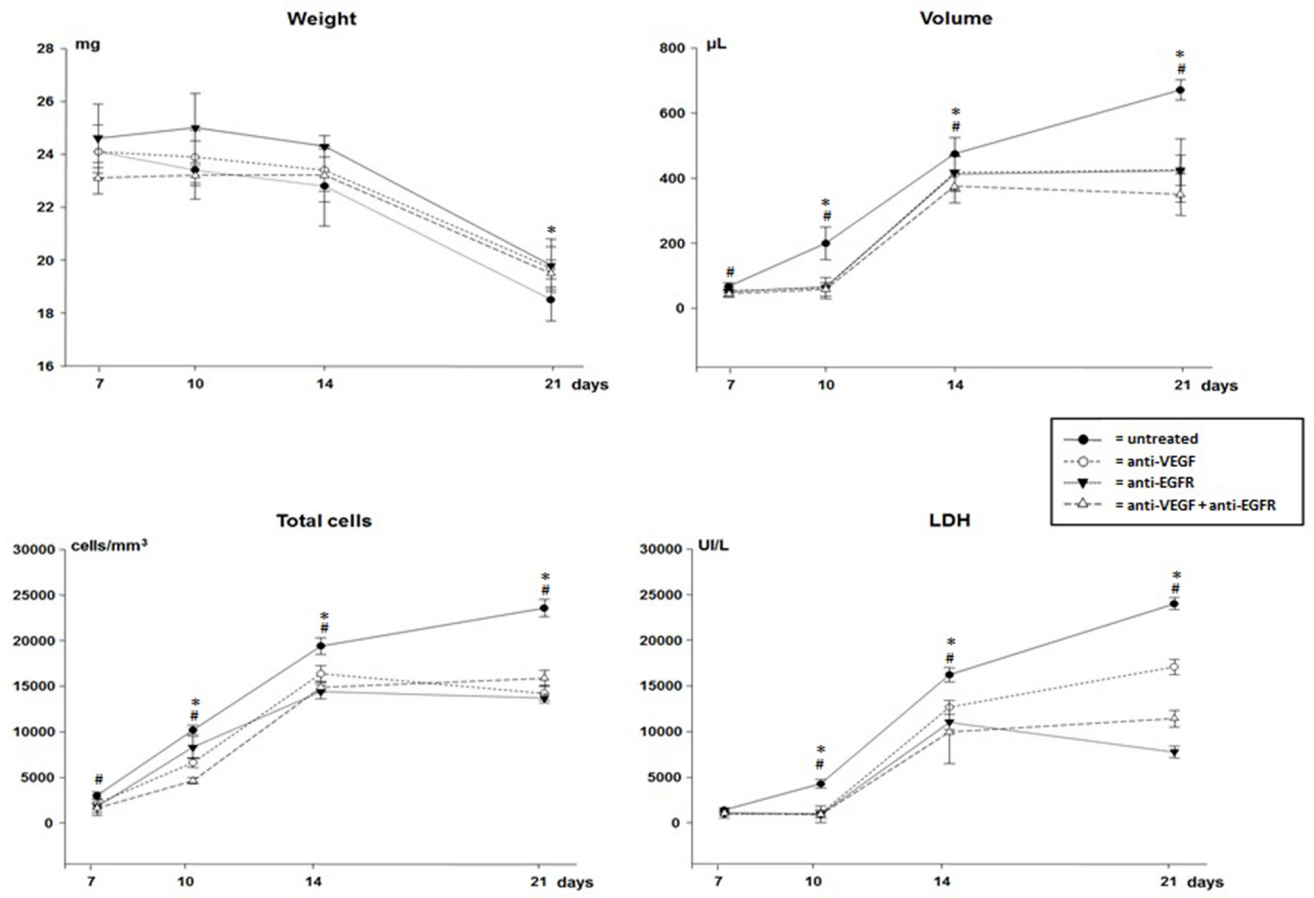
Weight, volume, total cells count and lactate dehydrogenase (LDH) of malignant pleural effusion of mice injected with LLC cells treated with anti-VEGF and/or anti-EGFR and untreated ^*^p<0.05 when compared by temporal evaluation and ^#^ p<0.05 when compared the groups.

All animals developed pleural cancer with malignant effusion (LLC cell implantation) and presented progressive weight loss without significant difference between treated and untreated animals (Figure 1).

There was progressive reduction of activity/mobility without statistical difference between the groups up to the 21^st^ day of the disease (score 3). However, animals treated with anti-EGFR and anti-VEGF+anti-EGFR on the 10^th^ (score 0) and on the 14^th^ day (score 1) were significantly more active than animals treated with anti-VEGF only (10 days - score 1 and 14 days - score 2; p <0.05) or those untreated.

**Figure 2:**
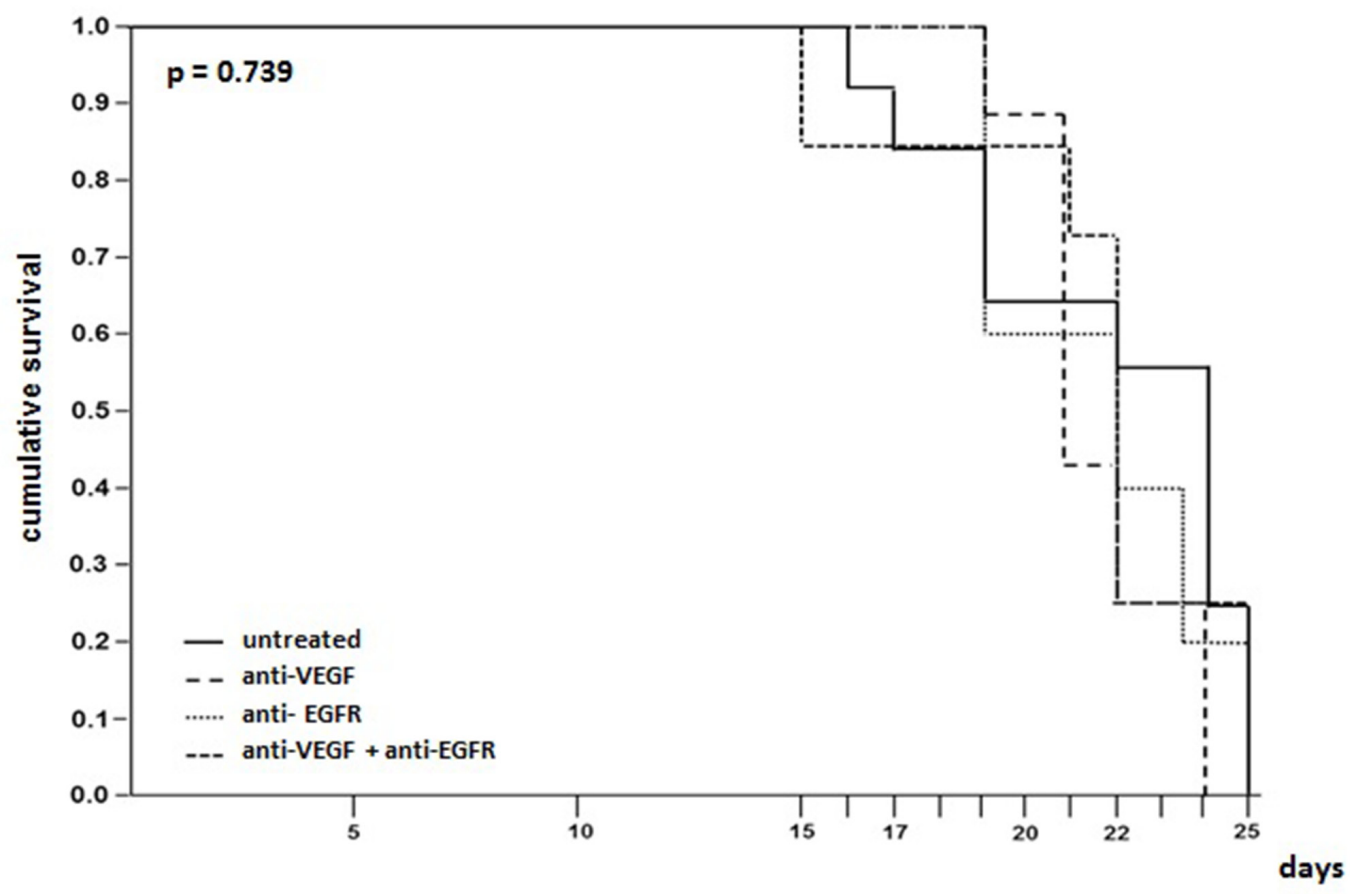
Survival time evaluation of mice injected with LLC cells treated with anti-VEGF and/or anti-EGFR and untreated

Pleural carcinomatosis was lethal in all groups with no difference between treated and untreated animals. In the group that received anti-VEGF the mortality was 100% on the 24^th^ day while the other groups showed maximum survival of 25 days (Figure 2).

All animals developed pleural tumors with masses and hemorrhagic pleural effusion evident. In the temporal evaluation there was a progressive increase in the volume of pleural fluid, especially in the untreated animals (Figure 1). However, the volume was significantly lower in all animals receiving treatment (p<0.05).

In the pleural fluid a progressive increase in cellularity was observed according to the evolution of malignant pleural disease. In all groups of treated animals cytological analysis showed significantly lower total cellularity than in untreated animals at all times analyzed (Figure 1). Malignant pleural effusion presented a mixed inflammatory infiltrate and macrophages interspersed with malignant cells.

On the 14^th^ day of the experiment there was a lower proportion of leukocytes in the pleural fluid of the mice treated with anti-VEGF+anti-EGFR (42±23 vs. 55±9 without treatment, 63±13 anti-VEGF and 63±5 anti-EGFR). However, except for a decrease in leukocytes and an increase of macrophages in the pleural fluid of untreated animals on the 21st day (58±7 vs. 35±6 anti-VEGF, 27±6 anti-EGFR and 32±2 anti-VEGF+anti-EGFR), there were no other significant differences in the differential cytological analyses.

As the malignant pleural disease progressed a significant increase in lactic dehydrogenase (LDH), proteins, VEGF and IL-6 levels in the pleural fluid of all animals was observed. With the exception of day 7, LDH levels were significantly lower in all treated animals (Figure 1), but no differences in protein levels were observed when comparing groups.

**Figure 3:**
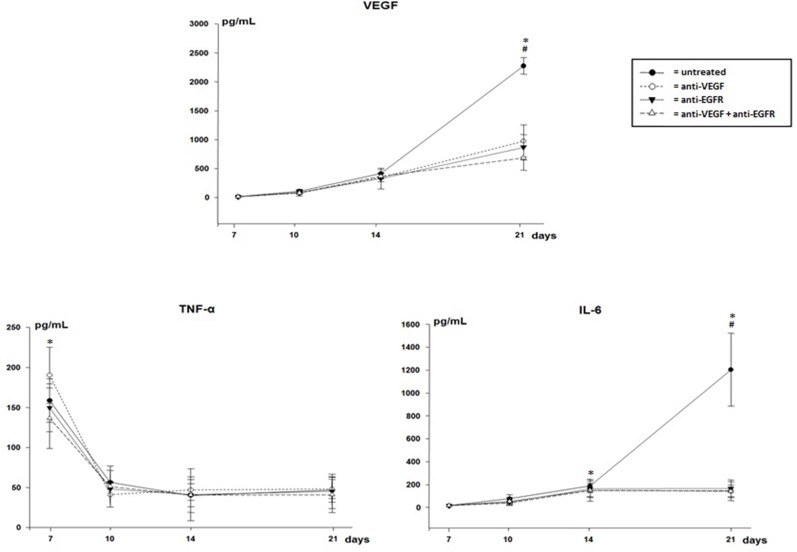
VEGF, TNF-α and IL-6 in pleural effusion of mice injected with LLC cells treated with anti-VEGF and/or anti-EGFR and untreated ^*^p<0.05 when compared by temporal evaluation and ^#^ p<0.05 when compared the groups.

The levels of VEGF and IL-6 in the pleural fluid were significantly higher on the 21^st^ day in untreated animals. However, TNF-α levels were higher, with peak production on the 7^th^ day and a decrease at all other times in all groups (Figure 3).

Though the method used, in which the detected level is above 3.9 pg/mL, EGF levels were undetectable in pleural fluid.

**Figure 4:**
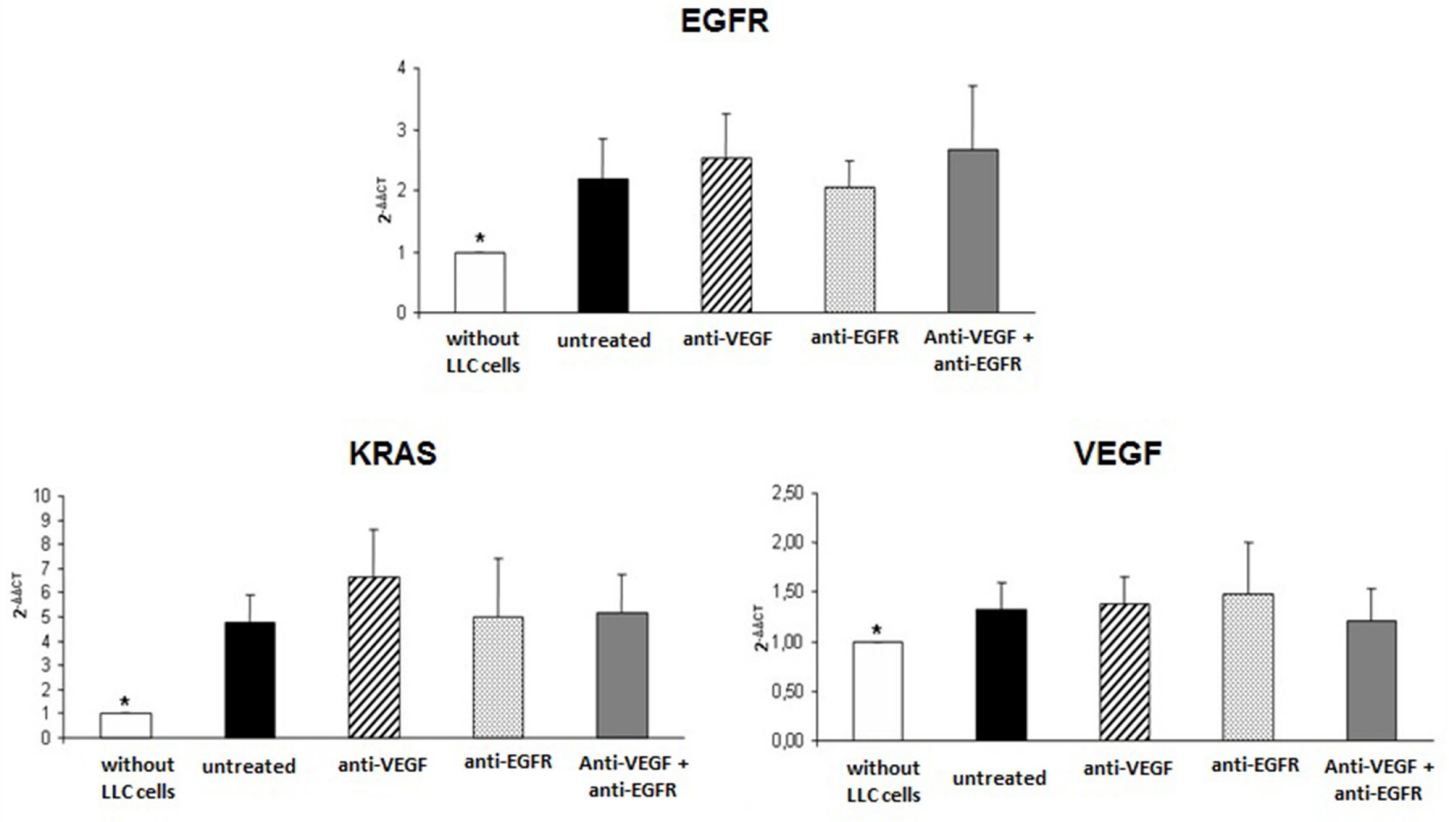
Gene expression of EGFR, KRAS and VEGF in tumor of mice injected with LLC cells treated with anti-VEGF and/or anti-EGFR and untreated ^*^p<0.05 when compared normal mice tissue with groups of the study.

In the evaluation of the gene expression of VEGF in tumor implants of treated and untreated animals there was overexpression of about 30% of VEGF when compared to the lungs of mice not submitted to induction of neoplasia. However no difference was observed in the gene expression of VEGF when comparing the groups of treated or untreated animals (Figure 4).

Similarly, EGFR and KRAS were overexpressed in treated and untreated tumors with no differences between groups (Figure 4), and with approximately twice the EGFR expression and approximately five times higher KRAS expression than in the mice not submitted to neoplasia induction.

For evaluation of EGFR and KRAS mutations we used tissue adjacent to the implants, LLC cells in culture and tumor implants. In the adjacent tissues no mutations were observed in the EGFR gene exons 18 to 22 and exon 2 of the KRAS gene. No sequencing mutations were observed for EGFR exons 18 to 22 in tumor tissue or cells in culture.

**Figure 5:**
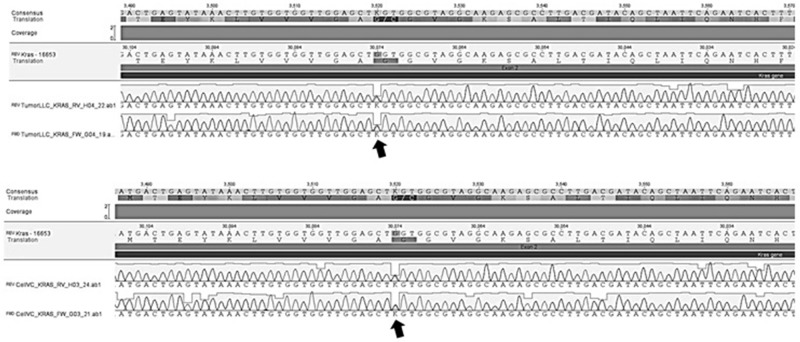
Genetic sequencing showing mutation (arrows) in exon 2 of KRAS gene in culture of LLC cells and tumor tissue of mice after LLC cells implantation

Exon 2 of the KRAS gene was identified as a mutation in both cultured and tumor cells extracted from mice after induction of neoplasia (Figure 5).

## DISCUSSION

In our study we used the model developed by Stathopoulos et al [18]. and modified by Acencio and colleagues [19] to evaluate the impact of targeted therapies (anti-VEGF and anti-EGFR) administered intrapleurally either alone or combined.

Isolated or combined therapies may inhibit different signaling pathways; combined therapies may have the potential to be more effective than inhibiting a single pathway and overcoming tumor resistance.

VEGF and EGFR inhibitors are key therapies in various types of tumors; there are close relationships between these two factors. Signaling of VEGF is regulated positively by EGFR expression and at the same time the positive regulation of VEGF independent of EGFR signaling, seems to contribute to resistance to EGFR inhibition. When both pathways are blocked this could improve antitumor efficacy and overcome resistance to EGFR inhibition, and may be further optimized when administered directly into the pleural cavity [20].

In this study using isolated and combined anti-VEGF and anti-EGFR therapies, Lewis cell-mediated pleural carcinomatosis was lethal in all groups.

According to the progression of malignant pleural disease there was progressive weight loss and reduction of activity. However, the animals treated with anti-EGFR or anti-VEGF+anti-EGFR were significantly more active in the intermediate phase of the disease (10^th^ and 14^th^ day).

In the assessment of pleural fluid there was an evident progressive increase in volume in all animals from the 14^th^ day of evolution of the disease. Larger volumes were detected in the untreated animals, demonstrating the action of these growth factors (VEGF and EGF) in the formation of malignant pleural effusion. Ribeiro and colleagues [21] demonstrated the participation of anti-VEGF in the formation of pleural effusion in animals with normal pleura with fluid induced by irritant agent. They observed the participation of anti-VEGF in angiogenesis, in the reduction of vascular permeability, and in the inhibition of inflammatory mediators.

Both VEGF and EGF are endothelial cell signaling factors and induce angiogenesis, acting directly and indirectly in mediating or producing inflammatory mediators [21, 20, 22].

An increase in the cellularity, LHD, total proteins, VEGF and IL-6 in the pleural fluid was observed according to the progression of the pleural malignancy; treated animals presented lower levels in their pleural fluid. These data demonstrate the action of these intrapleural therapies in the attempt to control malignant effusion, although there is no improvement in survival time after treatment.

Gene overexpression of VEGF, EGFR and KRAS was also observed. Many mechanisms have been proposed to explain this phenomenon and there is evidence to suggest that increased VEGF expression plays a role in resistance to anti-EGFR therapy [23–25]. Viloria-Petit and colleagues have observed the acquired resistance of tumor cells to anti-EGFR monoclonal antibodies associated with increased levels of VEGF, accompanied by an increase in angiogenic potential *in vitro* and tumor angiogenesis *in vivo* [26].

EGFR, a tyrosine kinase receptor considered to be oncogenic, is responsible for growth, survival, proliferation and differentiation of several cell types. Its activation occurs either through EGF or, in cases of mutations, by the activation of the tyrosine-receptor kinase by other mediators initiating multiple cascades of intracellular events [8, 20, 27]. When altered, either by hyperexpression, amplification or mutations, it induces uncontrolled growth or malignant phenotype, since this pathway physiologically regulates aspects of cell proliferation and survival [1, 20, 27]. In our study there was tumor overexpression of EGFR (2 times more) and KRAS (5 times more) compared to tumor-free lungs.

The KRAS gene is a key element in the EGF-mediated signaling pathway, regulating cell growth, differentiation, and apoptosis through interaction with multiple effectors [28].

We analyzed the presence of mutations in exons 18 to 22 of EGFR and of exon 2 of KRAS in tumors, where the mutation of the KRAS gene was detected both in LLC cell culture and in the tumor implants extracted from the mice.

Several studies have shown that mutations of EGFR and KRAS are mutually exclusive, suggesting that they have functionally equivalent roles in lung tumorigenesis. [29, 30] Similar to EGFR mutations, KRAS mutations also appear to be associated with distinct clinical and pathological features and vary according to tumor histology, ethnicity, and smoking history [31]. KRAS mutations occur most frequently in lung adenocarcinomas and less frequently in the squamous cell carcinoma subtype [32].

In contrast, although KRAS mutations have been identified in NSCLC tumors for more than 20 years, we are only beginning to understand their clinical significance.

Progress in this field has been hampered by relatively small studies with different methods of molecular analysis and by heterogeneity in histological subtypes, staging, administered treatment and survival criteria used. The clinical relevance of the KRAS mutational state in patients with NSCLC was assessed in one meta-analysis of 1,335 Caucasian and Asian patients who were included in 22 studies and were treated with gefitinib or erlotinib [33]. Despite the heterogeneity of the sample, pooled results suggest that KRAS mutations act as a negative predictive marker for tumor response in NSCLC patients treated with anti-EGFR therapies. Novel strategies for the treatment of KRAS mutated NSCLC tumors are required.

In this study we demonstrated that tumors from the LLC cells present KRAS mutation with tumor overexpression of VEGF, EGFR and KRAS. To the best of our knowledge, this study is the first in the literature describing the genetic characteristics and KRAS mutation in tumors originating from LLC cells. These findings indicate a more aggressive malignant phenotype tumor line, with uncontrolled growth and loss of apoptosis, associated with a worse prognosis and a lower response to EGFR inhibitor drugs.

Drugs administered intrapleurally can reduce volume and inflammatory mediators in pleural fluid. In addition anti-EGFR and the combination of anti-VEGF+anti-EGFR in the intrapleural space decreased morbidity, with significantly more active animals in the intermediate phase of the disease; there was no impact on survival. More studies should be done with targeted therapies geared toward tumoral genetic changes.

## MATERIALS AND METHODS

### Cell culture

The Lewis Lung Carcinoma (LLC) cells were purchased from the American Type Culture Collection (Manassas, VA) and were cultured at 37°C in 5% CO_2_ −95% air using Dulbecco's modified Eagle's medium (DMEM) with 10% fetal bovine serum.

### Animal model

Two hundred and five male (6-8 week old) C57BL/6 mice were obtained from the University of São Paulo/School of Medicine - Laboratory Animal Center. All animal care and experimental procedures were approved by the University Ethics Committee (CEUA/CAPPesq).

Animals were anesthetized using 35 mg/kg of ketamine hydrochloride (Cristalia, Brazil) and 5 mg/kg of xylazine hydrochloride (Bayer, Brazil) prior to all procedures. The intrapleural injection of 0.5 × 10^5^ LLC cells was performed according to previous methodology [24].

Four groups of 50 mice each received treatment with anti-VEGF (15mg/kg), anti-EGFR (400mg/m^2^), anti-VEGF+anti-EGFR or saline at 3, 7, 10 and 14 days. These animals were subdivided into two study groups; the first consisted of four sets of 40 animals each, which were euthanized after 7, 10, 14 or 21 days; the second group (four sets of 10 animals) was evaluated for survival expectancy.

The mice were euthanized according to the study calendar; the abdominal wall was opened and the viscera were retracted to visualize the diaphragm. Pleural fluid, when present, was gently aspirated and the volume was measured and placed in tubes for posterior evaluation.

As a standard reference value to gene expression evaluation we used five lung´s mice without injection of LLC cells and no treatment.

### Weight, mobility and survival analysis

After the inoculation procedure all animals were observed until complete recovery and they were evaluated for weight (g) and mobility by a subjective score of 0-3 (0= normal and 3 = stillness). We monitored mortality daily for all groups to obtain the survival curve.

### Biochemical assays

Lactic dehydrogenase (kinetic UV method) and total protein (Biuret method) were quantified in the pleural fluid using commercial kits (Wienner, Argentina) and analyzed in a semi-automatic device.

### Cytology

Pleural fluid cells were counted in a Neubauer chamber. After centrifugation, cells were prepared and the slides were stained using Leishman to determine the cell differential.

### Cytokine analysis

For Interleukin-6 (IL-6), Tumor Necrosis Factor-alpha (TNF-α), EGF and VEGF analysis (R&D System, Minneapolis, USA), samples of pleural effusion were collected at the same time points in EDTA tubes, centrifuged and the supernatant was removed and stored for later determination. Cytokine levels were measured by Enzyme-Linked Immunoabsorbent Assay (ELISA) according to the protocol suggested by the manufacturer. Minimum detection levels for IL-6 and VEGF were 15.6 pg/mL, TNF-α was 31.25 pg/mL and EGF was 3.9 pg/mL.

### Gene expression

#### RNA extraction and cDNA preparation

Total RNA was extracted from all tissue samples using the *RNeasy mini kit* from Qiagen, (Qiagen, Hilden, Germany) according to the instructions of the manufacturer. Purified RNA was diluted in 100 ng/μL of RNase-free H_2_O. cDNA was synthesized from total RNA following the *SuperScript III First-Strand Synthesis SuperMix kit* (Invitrogen, California/USA) instructions.

#### Quantitative polymerase chain reaction (PCR)

Expressions of EGFR, VEGF, KRAS and the reference gene GAPDH were quantified using the *Gene Amp 7500 Sequence Detection System* (Applied Biosystems, California, USA).

The real-time PCR was performed using the *SYBR Green I Master Mix Buffer* following manufacturer's instructions. Primer sequences are shown in Table 1. All primers were purchased from Life Technologies, California, USA.

**Table 1:** Sequence primers used by a RT PCR

Gene	*Primer forward*	*Primer reverse*
**EGFR**	TTGGCCTATTCATGCGAAGAC	GTCATGAGCCCTTCCACAAT
**VEGF**	TCGGCTGTCCATGAAAGTGA	TTGCAGGCGAGCCATCTT
**KRAS**	CAAGAGCGCCTTGACGATACA	CCAAGAGACAGGTTTCTCCATC
**GAPDH**	GGGTGTGAACCACGAGAAAT	GRCATGAGCCCTTCCACAAT

#### Sanger sequence for mutation evaluation

Direct sequencing of exons 18 to 22 from EGFR (NM_207655) and exon 2 from KRAS (NM_021284) was performed to identify possible genetic alteration related to lung carcinoma in the LLC cells.

Genomic DNA from LLC cells, tumors and adjacent tissue were purified using DNeasy Blood & Tissue Kit (Qiagen, Hilden, Germany) according to the manufacturer's instructions.

Samples were amplified using GoTaq® Flexi DNA Polymerase (Promega, Wisconsin, USA). For PCR and sequencing primers refer to Table 2. PCR products were cleaned up using ExoSAP-IT (Affimetrix, California, USA) and sequenced in both directions using BigDye® Terminator v3.1 Cycle Sequencing Kit (Applied Biosystems, California, USA). Sequencing reactions were run on a 3500xL Genetic Analyser (Applied Biosystems, California, USA) and were analyzed with Geneious R9 [34].

**Table 2:** *Primers* used for PCRs and sequencing

*Gene*	*Exon*	*Primer forward*	*Primer reverse*
	18	GGAAGTGGGGCTTTCTGTTG	AGTTCTGAGTAAGGATGGCAGT
	19	CTACCCAATTTTGAGATCACCGT	AGTAGCCCTTCACACCATGT
*EGFR*	20	CATGCAACATCCCAAAGGAGT	TCTCTTAGATCATCCTTGCTGCT
	21	TTGGTGTTGAGCAGCCTAGA	CCCCACTCAGAATCTTTGGC
	22	AGTGAGAGGTTCACAGCCTT	TTCAGTAGATGGACACGCTCA
*KRAS*	2	CATCTGTAGTCACTGAATTCGGA	CCTTGGAACTAAAGGACATCACA

### Statistics

The results are presented as mean and standard deviation. The levels of gene expression were analyzed using the 2^−ΔΔCT^ method [35]. Comparisons among the groups were performed using ANOVA followed by the comparison multiple test. For the survival time, Kaplan–Meier curves were established for each group and the times were compared using a log-rank test. A value of p<0.05 was considered significant. SigmaStat 3.1 (Systat, CA, USA) was used for the analyses.
